# Dietary fatty acid intake is associated with paraoxonase 1 activity in a cohort-based analysis of 1,548 subjects

**DOI:** 10.1186/1476-511X-12-183

**Published:** 2013-12-12

**Authors:** Daniel Seung Kim, Sean K Maden, Amber A Burt, Jane E Ranchalis, Clement E Furlong, Gail P Jarvik

**Affiliations:** 1 Department of Medicine, Division of Medical Genetics, University of Washington School of Medicine, Box 357720, Seattle, WA 98195-7720, USA; 2 Department of Genome Sciences, University of Washington School of Medicine, Seattle, WA, USA

**Keywords:** Paraoxonase 1, Dietary fatty acid intake, Saturated fats, Monounsaturated fats, Polyunsaturated fats, ω-3 fatty acids, Cardiovascular disease

## Abstract

**Background:**

Paraoxonase 1 (PON1) is a cardioprotective, HDL-associated glycoprotein enzyme with broad substrate specificity. Our previous work found associations between dietary cholesterol and vitamin C with PON1 activity. The goal of this study was to determine the effect of specific dietary fatty acid (DFA) intake on PON1 activity.

**Methods:**

1,548 participants with paraoxonase activity measures completed the Harvard Standardized Food Frequency Questionnaire to determine their daily nutrient intake over the past year. Eight saturated, 3 monounsaturated, and 6 polyunsaturated DFAs were measured by the questionnaire. To reduce the number of observations tested, only specific fatty acids that were not highly correlated (r < 0.8) with other DFAs or that were representative of other DFAs through high correlation within each respective group (saturated, monounsaturated, or polyunsaturated) were retained for analysis. Six specific DFA intakes – myristic acid (14 carbon atoms, no double bonds – 14:0), oleic acid (18:1), gadoleic acid (20:1), α-linolenic acid (18:3), arachidonic acid (20:4), and eicosapentaenoic acid (20:5) – were carried forward to stepwise linear regression, which evaluated the effect of each specific DFA on covariate-adjusted PON1 enzyme activity.

**Results:**

Four of the 6 tested DFA intakes – myristic acid (p = 0.038), gadoleic acid (p = 6.68 × 10^-7^), arachidonic acid (p = 0.0007), and eicosapentaenoic acid (p = 0.013) - were independently associated with covariate-adjusted PON1 enzyme activity. Myristic acid, a saturated fat, and gadoleic acid, a monounsaturated fat, were both positively associated with PON1 activity. Both of the tested polyunsaturated fats, arachidonic acid and eicosapentaenoic acid, were negatively associated with PON1 activity.

**Conclusions:**

This study presents the largest cohort-based analysis of the relationship between dietary lipids and PON1 enzyme activity. Further research is necessary to elucidate and understand the specific biological mechanisms, whether direct or regulatory, through which DFAs affect PON1 activity.

## Background

The beneficial effects of high-density lipoprotein (HDL) on cardiovascular health have recently come under increased scrutiny after both a large randomized clinical trial [1] and separate Mendelian randomization study [2] failed to show cardioprotective effects from raising HDL cholesterol levels (HDL-C) alone. Instead, focus has shifted to the atherosclerosis-related aspects of HDL biology that are not reflected in HDL-C measurements, such as paraoxonase 1 (PON1) activity.

PON1 is a glycoprotein enzyme with broad substrate specificity [3], which is at least partly responsible for the inhibitory and cardioprotective effects of HDL on lipid peroxidation and its resulting atherogenesis [4]. In addition to its cardioprotective effects, PON1 is protective against exposure to toxic organophosphorus (OP) compounds [5]. The activity of PON1 is measured by its catalytic efficiency for the hydrolysis of the substrates paraoxon (POase), diazoxon (DZOase), and phenylacetate (AREase). Of these measurements of PON1 activity, AREase is the most correlated with protein levels, as it is not drastically affected by the *PON1*_*Q192R*_ coding polymorphism [6,7].

Four well-known human polymorphisms affect PON1 activity: *PON1*_*C-108T*_ (rs705379)*, PON1*_*G-162A*_ (rs705381)*, PON1*_*M55L*_ (rs854560), and *PON1*_*Q192R*_ (rs662). Of these, *PON1*_*C-108T*_ has the largest effect on PON1 AREase activity due its promoter-altering properties [8-11], accounting for approximately 15% of PON1 AREase variance [12]. The *PON1*_*Q192R*_ polymorphism [13] is the primary determinant of toxic OP compound catalysis, accounting for over 65% of PON1 POase activity [12]. Rare protein-truncating and missense mutations in *PON1* have been identified and associated with PON1 activity [14,15].

Numerous environmental factors, including diet, have been associated with differential PON1 activity [16,17]. However, while dietary cholesterol is associated with PON1 activity in humans [18], the relationship between dietary fatty acid (DFA) intake and PON1 remains unclear. For example, rats fed a diet rich in oleic acid, a monounsaturated DFA (a fatty acid containing only a single double bond in its carbon chain) found in olive oil, had increased PON1 activity (+46%); however, when the rats were switched to a diet high in polyunsaturated, ω-3 and ω-6 DFAs, there was a significant decrease in PON1 activity (−39%) [19]. Similarly, human studies have found an increase in PON1 activity in 14 diabetic patients after meals rich in thermally stressed olive oil, with the effect greater in females than males [20]. Similarly, oleic acid intake, as determined from a 12-hour food recall survey, was found to be associated with increased PON1 activity, although the effect was only significant in subjects with the homozygous “RR” genotype at *PON1*_*Q192R*_[21]. Finally, a decrease in PON1 activity in both healthy men and women when switching from a diet rich in saturated fats to one composed primarily of trans DFAs has been reported [22].

Research into the *in vitro* interaction of PON1 and DFAs have similarly presented conflicting results. Negatively charged lipids, including saturated, monounsaturated, and polyunsaturated DFAs, have all been reported to inhibit PON1 enzyme activity *in vitro*[23], with polyunsaturated fatty acids having the largest inhibitory effect [24]. However, monounsaturated fatty acids (and to a lesser extent, saturated fats) have also been shown to protect PON1 from ascorbate/copper-mediated oxidative inactivation [24,25]. Notably, polyunsaturated fats prevented this monounsaturated DFA-dependent oxidative protective effect [24]. In addition, monounsaturated fats have been reported to preserve PON1 enzyme activity during lengthy *in vitro* incubation periods [24]*.*

The Carotid Lesion Epidemiology and Risk (CLEAR) cohort is a Seattle-based carotid artery disease (CAAD) case–control cohort, comprised primarily of veterans, collected to identify risk factors for CAAD, CAAD progression, and other atherosclerotic disease end-points. Previous work in the CLEAR cohort has identified novel dietary factors – vitamins C and E [26], cholesterol intake [18], and dietary iron in non-anemic subjects [18] – which are associated with PON1 enzyme activity. The majority of human studies examining the relationship between DFAs and PON1 have been small (n < 100 subjects), and the *in vitro* evidence conflicting. Thus, the goal of the present study was to evaluate the effects of specific DFA intakes on PON1 activity as measured by AREase within this cohort of 1548 subjects, to elucidate the relationship of fatty acids and PON1.

## Methods

### Ethics statement

Institutional review boards at the University of Washington, Virginia Mason Medical Center, and Veterans Affairs Puget Sound approved the CLEAR study. Written, informed consent was obtained from each participant of the study.

### Sample

The study population for this analysis consisted of 1,548 participants from the previously described CLEAR study [27,28]. The cohort consisted of 380 participants with CAAD as determined by ultrasound (>50% stenosis in either carotid artery), 73 participants with moderate obstruction (15-49% obstruction in at least one carotid artery), 96 subjects with other phenotypes, including peripheral artery disease (PAD) and coronary artery disease (CHD), and 999 controls (<15% carotid stenosis bilaterally and absence of PAD and CHD). Current smoking status and reported ancestry were obtained by self-report. Insulin use was determined via self-report matched to hospital pharmacy records. Exclusion criteria included familial hypercholesterolemia, total fasting cholesterol greater than 400 mg/dl, hypocoagulable state and/or the use of anticoagulant medication, post-organ transplant, or the inability to consent. Descriptive statistics of the studied subset of the CLEAR study are presented in Table 1.

**Table 1:** Baseline characteristics of the studied subset of the CLEAR cohort

**Baseline characteristics**	**CLEAR cohort (N = 1548)**
Ethnicity, n (%)	
European ancestry, not Hispanic	1240 (80.1)
Hispanic ancestry	36 (2.3)
African ancestry	128 (8.3)
Asian/Pacific Islander ancestry	144 (9.3)
Gender, n (%)	
Female	544 (35.1)
Male	1004 (64.9)
Age, mean ± SD, years	64.84 ± 9.69
Current smoker, n (%)	179 (11.6)
Dietary fat intake	
Saturated fats	
ln(Myristic acid (14:0) intake), mean ± SD, g/day	1.03 ± 0.339
Monounsaturated fats	
ln(Oleic acid (18:1) intake), mean ± SD, g/day	3.14 ± 0.419
ln(Gadoleic acid (20:1) intake), mean ± SD, g/day	0.242 ± 0.143
Polyunsaturated fats	
ln(α-Linolenic acid (18:3) intake), mean ± SD, g/day	0.795 ± 0.239
ln(Arachidonic acid (20:4) intake), mean ± SD, g/day	0.140 ± 0.0666
ln(Eicosapentaenoic acid (20:5) intake), mean ± SD, g/day	0.138 ± 0.123
PON1 AREase activity, mean ± SD, IU	149.58 ± 50.47

### PON1 genotyping and phenotyping

The four *PON1* polymorphisms with the largest effects on PON1 enzyme activity, *PON1*_*C-108T*_*, PON1*_*G-162A*_*, PON1*_*M55L*_*,* and *PON1*_*Q192R*_, were genotyped using previously described methods [8,9]. PON1 activity was measured by the rate of enzymatic degradation of phenylacetate (AREase) via a continuous spectrophotometric assay with lithium heparin plasma, as AREase is least affected by the *PON1*_*Q192R*_ polymorphism and also is more closely related to PON1 protein levels [6,7]. PON1 AREase activity was measured in triplicate and averaged for analysis. Measurements were made blinded to phenotype and diet data.

### Food-frequency questionnaire

At enrollment, participants were asked to complete the standardized Harvard food frequency questionnaire developed by the Health Professionals Follow-Up Study (https://regepi.bwh.harvard.edu/health/nutrition.html). The survey asked participants for their average frequency of intake of specified portions of 131 foods, vitamins, and mineral supplements. The surveys were then returned to Harvard School of Public Health and the Brigham and Women’s Hospital, where they underwent quantitative analysis to return the inferred average intake of all micronutrients, including DFAs, vitamins, and minerals. The Harvard Food Frequency Questionnaire has been validated against two, in-depth, 1-week diet records taken approximately six months apart [29]. Additionally, the inferred intake of dietary fats have been validated against plasma lipid measurements [30,31].

### DFA selection

**Figure 1:**
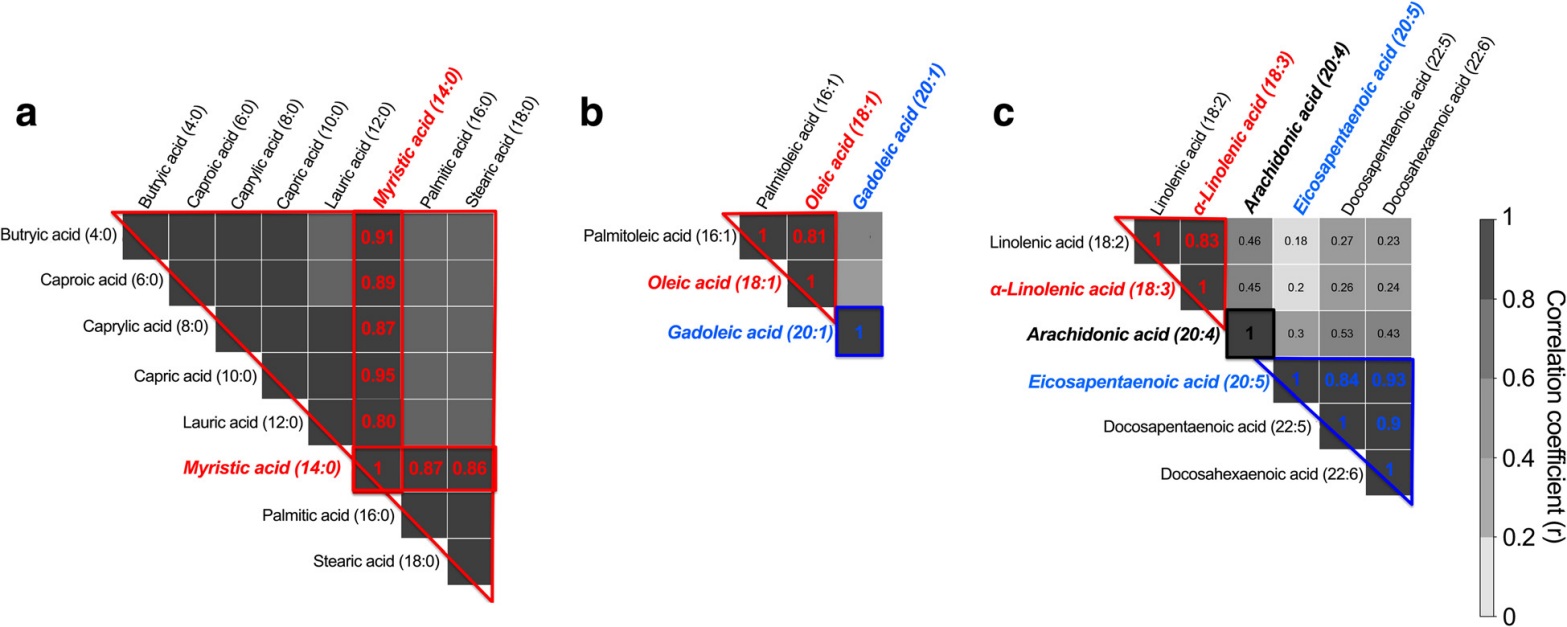
**Selection of specific dietary fatty acid intakes for analysis by correlation.** Correlation matrix between specific dietary fatty acids within the group of saturated **(a)**, monounsaturated **(b)**, and polyunsaturated fatty acids **(c)**. To reduce the number of statistical tests, only those dietary fatty acids not highly correlated (Pearson’s pairwise correlation coefficient, r, < 0.8) with others in its group were carried forward to analysis. If numerous fatty acid intakes were highly correlated (r ≥ 0.8), a single representative was chosen.

To reduce the number of statistical tests, only DFAs not highly correlated (r < 0.8) with other DFAs or that were representative of other DFAs through high correlation within each fatty acid group (saturated, monounsaturated, and polyunsaturated) were carried forward to statistical testing, as presented in Figure 1. Linear correlation within each fatty acid group was assessed by Pearson’s correlation coefficient (r). Of the 8 saturated DFAs measured by the food frequency questionnaire, all were highly correlated with myristic acid, composed of 14 carbons and no double bonds (14:0). Within the monounsaturated (fatty acids with one double bond) fatty acid group, palmitoleic (16:1) and oleic acid (18:1) were highly correlated with each other, while the third member, gadoleic acid (20:1) was not strongly correlated with either. Due to prior reports in the literature of a positive association between oleic acid and PON1 activity [19,20,24], oleic and gadoleic acid were carried forward to statistical testing. Within the polyunsaturated fat group, there were 3 clusters of correlation (see Figure 1). From these correlation blocks, α-linolenic acid (18:3, a ω-3 DFA), arachidonic acid (20:4, a ω-6 DFA), and eicosapentaenoic acid (20:5, a ω-3 DFA) moved on to the stepwise linear regression procedure.

### Statistical methods

Natural log transformation was performed for each of the 6 specific DFA intake variables, as they all displayed a skewed distribution. Extreme observations were Winsorized to 3 standard deviations from the mean [32]. For food frequency data, participants were excluded if their caloric intake was <800 calories/day or >4000 calories/day. Additionally, participants were excluded if the returned survey had ≥70 missing items.

All analyses were performed in R (http://www.r-project.org/). Genotypes were coded using an additive model. Stepwise linear regression was performed with the 6 specific DFA intake variables entering the model. Model comparison was performed using Akaike’s information criterion (AIC), beginning with a base model that included age, sex, current smoking status, self-reported race (with European ancestry as the reference group, as they comprised the majority of the cohort), and the genotypes of the 4 functional *PON1* polymorphisms as covariates for the prediction of PON1 AREase activity. Only specific DFA intakes that improved model prediction of the outcome PON1 AREase activity were retained in the final model. To identify whether DFAs account for variance previously explained by dietary cholesterol or other variables, a secondary analysis in the previously published subset of the cohort (n = 1402 participants) was performed; in addition to the effects of dietary cholesterol, vitamin C, folate, iron, and insulin use on PON1 activity that had previously been reported to be significant in this subset [18].

## Results

Demographic, clinical, and dietary fat intake variables are presented in Table 1. Participants of self-reported European, non-Hispanic ancestry composed the majority of the cohort (80.1%). Subjects of Asian (9.3%), African (8.3%), and Hispanic (2.3%) ancestry composed the remainder of the cohort. Males accounted for approximately two-thirds (64.9%) of the studied population. The average age of all subjects was 64.8 years, and 11.6% of the cohort were current smokers. PON1 AREase activity had a mean of 149.6 IU with a standard deviation of 50.5 forming an approximate normal distribution.

To reduce the number of statistical tests performed and problems with colinearity, only 6 of the 17 available DFA intakes were selected for stepwise linear regression. The 6 selected DFAs were highly correlated with other DFAs in each group (saturated, monounsaturated, polyunsaturated) and therefore captured the majority of the group variation while minimizing the problems that arise with colinearity. The 6 selected DFAs were: myristic acid (14:0, saturated fat), oleic acid (18:1, monounsaturated fat), gadoleic acid (20:1, monounsaturated fat), α-linolenic acid (18:3, polyunsaturated ω-3 fat), arachidonic acid (20:4, polyunsaturated ω-6 fat), and eicosapentaenoic acid (20:5, polyunsaturated ω-3 fat). The correlation between the selected DFAs and the other DFAs in each group are summarized in Figure 1.

A baseline regression model containing the 4 functional *PON1* variants (*PON1*_*C-108T*_*, PON1*_*G-162A*_*, PON1*_*M55L*_*,* and *PON1*_*Q192R*_), age, sex, current smoking status, and genetic ancestry explained 25.1% of PON1 AREase variance. We then examined a best-fit model using stepwise linear regression with the base model and the 6 DFAs identified through correlation testing. Only those DFAs that improved model prediction through assessment by AIC were retained in the final, best-fit regression model.

In the best-fit regression model, 4 of the 6 DFA intakes were retained in addition to the base model, together explaining 26.3% of PON1 AREase activity (see Table 2). The 4 specific DFAs (myristic acid (p = 0.038), gadoleic acid (p = 6.68 × 10^-7^), arachidonic acid (p = 0.00069), and eicosapentaenoic acid (p = 0.0134)) serially explained 0.25%, 0.58%, 0.61%, and 0.29% of PON1 AREase activity, respectively. Myristic acid (beta coefficient = 7.71), a saturated fat, and gadoleic acid (beta coefficient = 59.50), a monounsaturated fat, were both significantly positively associated with an increase in PON1 AREase activity. Conversely, dietary intake of the two polyunsaturated fats, arachidonic acid (beta coefficient = −67.15) and eicosapentaenoic acid (beta coefficient = −33.01), were both significantly negatively associated with PON1 AREase activity. Sensitivity analyses stratifying by sex and CAAD status did not reveal significant differences in subgroup coefficient sizes or p-values, suggesting that these factors were not affecting the relationship between specific DFA intake and PON1 enzyme activity.

**Table 2:** Best-fit model from stepwise linear regression predicting PON1 AREase activity using dietary fat intake variables (n = 1548 subjects)

**Variable**^**a,b**^	**Estimate ± SE**	**% PON1 AREase variation**	***P***
*(Intercept)*	236.85 ± 9.57	-	< 2 × 10^-16^
*PON1*_*C-108T*_	−26.48 ± 1.94	13.30%	< 2 × 10^-16^
*PON1*_*G-162A*_	−1.31 ± 2.24	0.21%	0.56
*PON1*_*Q192R*_	−12.34 ± 2.08	2.38%	3.83 × 10^-9^
*PON1*_*M55L*_	−6.16 ± 2.16	0.42%	0.0044
Age	−0.94 ± 0.12	2.81%	5.54 × 10^-15^
Sex	17.95 ± 2.42	4.70%	2.17 × 10^-13^
Current smoker	−13.43 ± 3.58	0.44%	0.00018
Hispanic ancestry^c^	2.43 ± 7.40	0.01%	0.74
African ancestry^c^	−17.04 ± 4.52	0.71%	0.00017
Asian ancestry^c^	−5.55 ± 4.278	0.13%	0.19
ln(Myristic acid (14:0) intake)	7.71 ± 3.71	0.25%	0.038
ln(Gadoleic acid (20:1) intake)	59.50 ± 11.92	0.58%	6.68 × 10^-7^
ln(Arachidonic acid (20:4) intake)	−67.15 ± 19.76	0.61%	0.00069
ln(Eicosapentaenoic acid (20:5) intake)	−33.01 ± 13.33	0.29%	0.013

^a^ln(Oleic acid (18:1) intake) was not significantly associated with PON1 AREase activity (p = 0.246, beta coefficient = 3.52) and was not retained in the final stepwise regression model.

^b^ln(α-Linolenic acid (18:3) intake) was not significantly associated with PON1 AREase activity (p = 0.913, beta coefficient = −0.579) and was not retained in the final stepwise regression model.

^c^Genetic ancestry coded as dummy variable, with European ancestry (the majority of the CLEAR cohort) subset used as the reference group.

To determine whether the PON1 activity–DFA association was related to the prior report of an association with dietary cholesterol in this cohort, we performed a secondary analysis in the previously published subset of the cohort (n = 1402 participants), in which an association between dietary factors including cholesterol and PON1 activity was detected [5,18]. Gadoleic (r = 0.33), myristic (r = 0.64) and arachidonic acid (r = 0.82) were significantly correlated (p < 0.001) with dietary cholesterol intake, while eicosapentaenoic acid (r = 0.16) was not.

**Figure 2:**
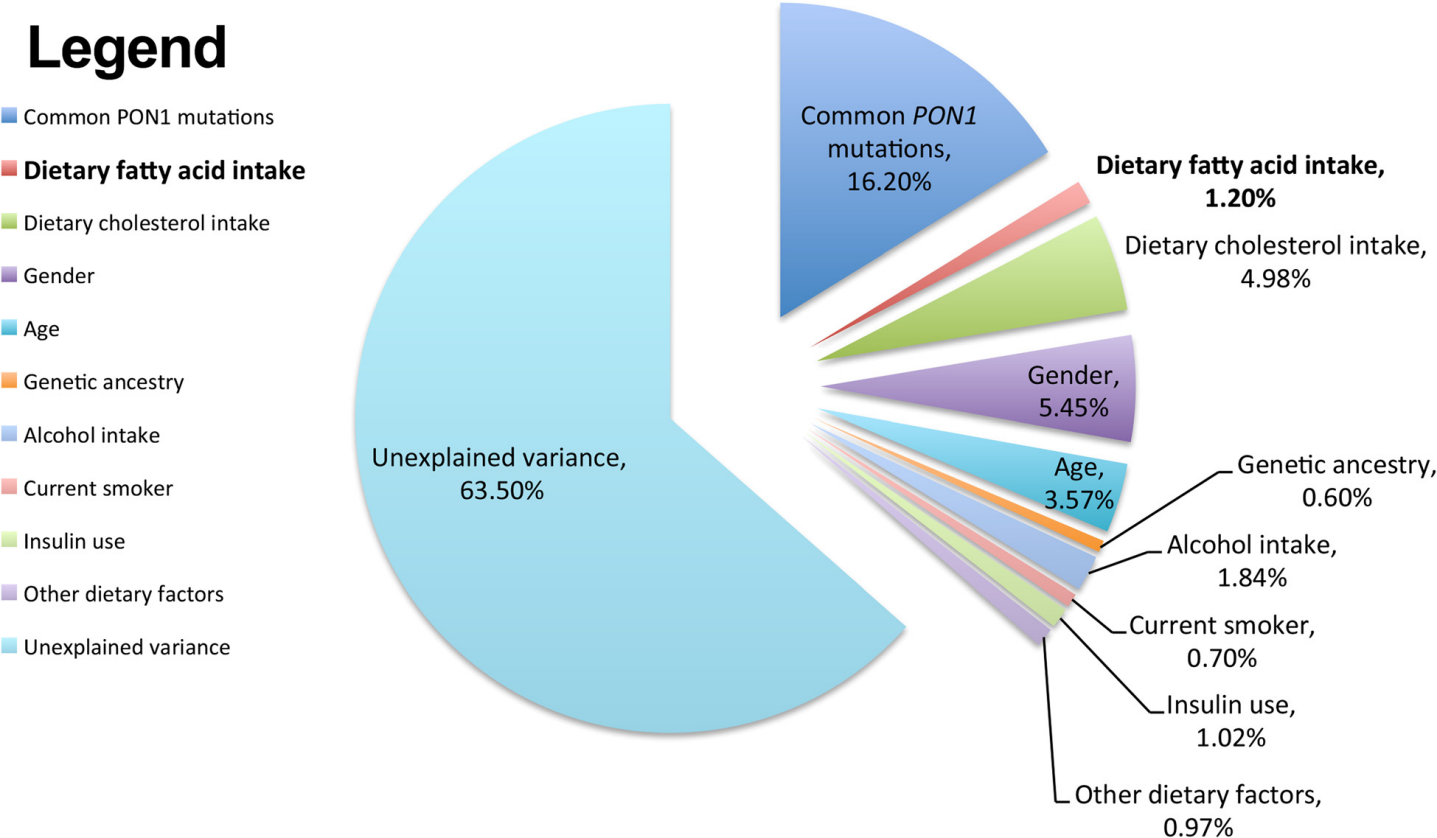
**Percentage of PON1 AREase activity explained by the dietary covariates considered.** Refer to Table 3 for complete stepwise model information.

In the best-fit model, 36.5% of PON1 AREase activity was explained, as opposed to 35.04% when DFAs were not included in the model (see Table 3 for specific model coefficients and p-values and Figure 2 for a summary of PON1 AREase activity explained). All 6 previously reported dietary and clinical variables that affected PON1 AREase activity (dietary cholesterol, vitamin C, folate, iron, alcohol intake, and insulin use) were retained in the final model in addition to the 4 specific DFA intakes. Addition of dietary DFA to the model reduced the coefficient of the dietary cholesterol effect by approximately 10% (from 59.70 to 56.41) and the variance explained by dietary cholesterol from 5.45 to 4.98%. Similarly, the effect sizes of gadoleic (from 59.50 to 46.78) and arachidonic acid (from −67.15 to −34.17) decreased by 21.4% and 49.1%, respectively, with dietary cholesterol intake in the model. However, DFAs accounted for 1.2% of PON1 activity variance; thus, the total variance explained by dietary cholesterol and DFAs was greater than for dietary cholesterol alone.

**Table 3:** Best-fit model from stepwise linear regression predicting PON1 AREase activity using both dietary fat and other intake variables (n = 1402 subjects)

**Variable**	**Estimate ± SE**	**% PON1 AREase variation**	***P***
*(Intercept)*	−71.40 ± 35.70	-	0.046
*PON1*_*C-108T*_	−27.99 ± 2.13	13.61%	<2 × 10^-16^
*PON1*_*G-162A*_	−2.42 ± 2.40	0.11%	0.31
*PON1*_*Q192R*_	−12.73 ± 2.24	1.92%	1.73 × 10^-8^
*PON1*_*M55L*_	−5.50 ± 2.31	0.54%	0.017
Age	−0.808 ± 0.135	3.57%	2.89 × 10^-9^
Sex	16.88 ± 2.77	5.45%	1.49 × 10^-9^
Current smoker	−15.34 ± 3.88	0.70%	8.24 × 10^-5^
Hispanic ancestry	6.06 ± 8.27	0.001%	0.46
African ancestry	−12.99 ± 5.02	0.58%	0.0097
Asian ancestry	−5.44 ± 5.29	0.015%	0.30?
Insulin use	−13.07 ± 5.76	1.02%	0.023
ln(Dietary cholesterol)^a,b,c^	53.41 ± 6.22	4.98%	<2 × 10^-16^
Alcohol category	6.27 ± 1.17	1.84%	1.11 × 10^-7^
ln(Vitamin C)	4.73 ± 1.51	0.23%	0.0018
Iron	−0.219 ± 0.0794	0.54%	0.0057
Folate	−0.00904 ± 0.00466	0.19%	0.053
ln(Myristic acid (14:0) intake)^a^	8.95 ± 4.05	0.41%	0.027
ln(Gadoleic acid (20:1) intake)^b^	46.78 ± 12.60	0.27%	0.00021
ln(Arachidonic acid (20:4) intake)^c^	−34.17 ± 21.56	0.15%	0.11
ln(Eicosapentaenoic acid (20:5) intake)	−36.96 ± 14.24	0.36%	0.0096

^a^ln(Dietary cholesterol) was significantly correlated (p < 0.001) with ln(Myristic acid) (r = 0.64).

^b^ln(Dietary cholesterol) was significantly correlated (p < 0.001) with ln(Gadoleic acid) (r = 0.33).

^c^ln(Dietary cholesterol) was significantly correlated (p < 0.001) with ln(Arachidonic acid) (r = 0.82).

## Discussion

PON1 is an HDL-associated enzyme that is involved in numerous human disease-related pathways. PON1 is atheroprotective [4,33], an antioxidant [34,35], can help to decrease the lethality of *Pseudomonas aeruginosa* infections [36,37], and it hydrolyzes numerous compounds – including pharmacologic agents [3,17,38] and toxic organophosphorus compounds [39,40]. A full understanding of PON1 should include the dietary factors that affect its expression and activity [18,26].

In the current study, we analyzed what we believe to be the largest cohort with both dietary intake and PON1 enzyme activity data, and report that the monounsaturated DFA, gadoleic acid (20:1), is strongly associated with an increase in PON1 activity. We also observed a strong decrease in PON1 enzyme activity associated with increasing polyunsaturated fat intake of arachidonic (20:4) and eicosaepentaenoic (20:5) acid. Finally, we report a proportionally smaller, though significant, effect of the saturated fat, myristic acid (14:0), on PON1 AREase activity. “We note that when considering previously reported dietary variables, the addition of DFA intake explained an additional 1.2% of PON1 AREase activity. Although 1.2% of PON1 activity may seem modest, it compares well to the scale of effects found for complex traits, including lipids [41]. Moreover, this data may reveal a new biological avenue for investigation regarding the potential regulation of PON1 by the dietary intake of fatty acids; namely, whether the mechanism through which DFAs intake affects PON1 activity is through gene regulation, direct protein interaction, or other more indirect processes. Finally, due to the ubiquitous nature of PON1 in human disease and physiology [17], understanding even a small portion of its variance is of high importance.

Monounsaturated fats have been previously positively associated with PON1 activity in human [20,21] and animal [19] studies. Evidence from *in vitro* studies suggests that both saturated and monounsaturated fats bind to a specific and protective site, separate from the catalytic active site, to prevent inactivation of PON1 through oxidation [24,25]. Moreover, *in vitro* evidence suggests that this binding of saturated and monounsaturated fats decreases PON1 activity only marginally (approximately 10%) [24]. The strongly protective effects of monounsaturated DFAs are broad: protection of PON1 from oxidation was not dependent on either carbon chain length or location of the double bond [24]. In our specific study, we did not find a strong association between oleic acid (the most commonly reported monounsaturated fat) and PON1 activity; when gadoleic acid was removed from the best-fit regression model, oleic acid was not significantly associated with PON1 activity (see Table 2). We did, however, find a strongly positive association of gadoleic acid (beta coefficient = 59.50, p = 6.68 × 10^-7^) on PON1 activity. The exact molecular mechanism for our finding, though suggested, is yet unknown, as are possible other mechanisms, including potential effects on *PON1* expression.

While the ω-3 and ω-6 DFAs commonly found in fish oil are generally thought to be cardioprotective [42], prior research has found fish oils, and the polyunsaturated fatty acids that compose fish oil, to be inhibitory to PON1 activity [19,23,24]. *In vitro* work suggests that polyunsaturated DFAs are recognized by the active site of PON1 and therefore act as competitive inhibitors of PON1 enzyme activity [24]. Moreover, binding of polyunsaturated fats appears to change the conformation of the protein, preventing protective binding of monounsaturated and saturated DFAs and, therefore, increasing the susceptibility of PON1 to inactivation from oxidation [24]. Consistent with prior report, we found that 2 (arachidonic and eicosaepentaenoic acid) of the 3 tested polyunsaturated DFAs were negatively associated with PON1 activity. The third polyunsaturated fatty acid, α-linolenic acid, trended negative, but was not significantly associated with PON1 activity (see Table 2). Thus, the cardioprotective effects of fish oil and polyunsaturated fats appear to occur in spite of what appear to be inhibitory effects on PON1 enzyme activity, whose activity is associated with atheroprotection.

When considering dietary intakes that we previously have reported [18], we were able to explain a total of 36.5% of PON1 AREase activity. However, we note that with the addition of these dietary covariates, there was a decrease in the magnitude of the beta coefficients and percentage of PON1 activity explained for both gadoleic acid and arachidonic acid. This likely is due to the highly significant correlation between both gadoleic (r = 0.33) and arachidonic (r = 0.82) acid with dietary cholesterol intake. Dietary cholesterol intake was the third most predictive covariate (after *PON1*_*C-108T*_ and gender) and accounted for approximately 5% of PON1 AREase activity. However, after accounting for the effects of dietary cholesterol intake, both gadoleic and arachidonic acid were retained in the best-fit stepwise regression model. This indicates that both DFAs have effects on PON1 activity that are independent of the large effects of dietary cholesterol intake and which aid in the prediction of PON1 AREase variance.

Strengths of this study include its large, well-characterized community-based cohort with dietary intake information, genetic data, and lipid phenotypes. To the best of the authors’ knowledge, this is the largest analysis of the effects of DFAs on PON1 activity. Limitations include the lack of ethnic diversity and that the cohort was collected primarily for CAAD, with participants that tended to be older than the general population. Together, these limitations may limit the generalizations and applications of these findings. Finally, the food frequency questionnaire used had limited data on several dietary covariates of interest: namely, trans fats, which have previously been reported to be inhibitory to PON1 activity [22]. As a result, some DFA associations with PON1 could not be assessed in this analysis.

In conclusion, our study has identified and confirmed the effects of specific fatty acid intakes on PON1 activity in a large cohort collected for vascular disease. Specifically, we report the positive association of saturated fats (myristic acid, p = 0.038) and monounsaturated fats (gadoleic acid, p = 6.68 × 10^-7^) on PON1 AREase activity and the PON1 activity decreasing effects of the polyunsaturated fats, arachidonic (p = 0.00069) and eicosapentaenoic (p = 0.013) acid. When considered in conjunction with our previously reported dietary, genetic, and clinical covariates [18], we were able to explain 36.5% of PON1 AREase activity. Further work characterizing the 63.5% unexplained PON1 variance should consider the possible effects of rare *PON1* variation [14,15], epistasis of *PON1* with other genes, and gene-by-environment interactions [43] in the attempts to further understand the determinants of this important and multi-faceted enzyme.

## Competing interest

The authors declare that they have no competing interests.

## Authors’ contributions

DSK, CEF, and GPJ came up with hypothesis and study design. JER performed laboratory analyses. DSK, SKM, and AAB performed statistical analyses. DSK, SKM, CEF, and GPJ wrote the manuscript. All authors read and approved the final manuscript.
